# Do patients with and survivors of COVID-19 benefit from telerehabilitation? A meta-analysis of randomized controlled trials

**DOI:** 10.3389/fpubh.2022.954754

**Published:** 2022-09-28

**Authors:** Jiapeng Huang, Ye Fan, Kehong Zhao, Chunlan Yang, Ziqi Zhao, Yin Chen, Jiaen Yang, Tingting Wang, Yun Qu

**Affiliations:** ^1^Department of Rehabilitation Medicine, West China Hospital, Sichuan University, Chengdu, China; ^2^Key Laboratory of Rehabilitation Medicine in Sichuan Province, West China Hospital, Sichuan University, Chengdu, China; ^3^Research Laboratory of Neurorehabilitation, Research Institute of Rehabilitation Medicine, West China Hospital, Sichuan University, Chengdu, China; ^4^Second Clinical Medical College, Guangzhou University of Chinese Medicine, Guangzhou, China; ^5^The Second Affiliated Hospital of Guangzhou University of Chinese Medicine, Guangdong Provincial Hospital of Chinese Medicine, Guangzhou, China; ^6^Department of Rehabilitation Medicine, Affiliated Foshan Gaoming Hospital of Guangdong Medical University, Guangdong, China

**Keywords:** telerehabilitation, COVID-19, physical function, psychological function, telemedicine, eHealth, meta-analysis

## Abstract

**Background:**

Coronavirus disease 2019 (COVID-19) significantly impacts physical, psychological, and social functioning and reduces quality of life, which may persist for at least 6 months. Given the fact that COVID-19 is a highly infectious disease and therefore healthcare facilities may be sources of contagion, new methods avoiding face-to-face contact between healthcare workers and patients are urgently needed. Telerehabilitation is the provision of rehabilitation services to patients at a distance *via* information and communication technologies. However, high-quality evidence of the efficacy of telerehabilitation for COVID-19 is still lacking. This meta-analysis aimed to investigate the efficacy of telerehabilitation for patients with and survivors of COVID-19.

**Methods:**

We searched the Cochrane Library, EMBASE, Medline (*via* PubMed), PEDro, ClinicalTrials.gov, and WHO International Clinical Trials Registry Platform from January 1st, 2020 to April 30th, 2022 for randomized controlled trials published in English, which aimed to evaluate the efficacy of telerehabilitation vs. face-to-face rehabilitation, usual care, or no treatment for COVID-19. Methodological quality and overall evidence quality of the included studies were assessed. The statistical reliability of the data was quantified using the trial sequential analysis.

**Results:**

Seven randomized controlled trials with eight comparisons were included and all of them were used for meta-analysis. The meta-analyses of absolute values showed the superiority of telerehabilitation over no treatment or usual care for dyspnea (Borg scale: mean difference = −1.88, −2.37 to −1.39; Multidimensional dyspnea-12: mean difference = −3.70, −5.93 to −1.48), limb muscle strength (mean difference = 3.29; 2.12 to 4.47), ambulation capacity (standardized mean difference = 0.88; 0.62 to 1.14), and depression (mean difference = −5.68; −8.62 to −2.74). Significant improvement in these variables persisted in the meta-analyses of change scores. No significant difference was found in anxiety and quality of life. No severe adverse events were reported in any of the included studies.

**Conclusions:**

Moderate- to very low-quality evidence demonstrates that telerehabilitation may be an effective and safe solution for patients with and survivors of COVID-19 in dyspnea, lower limb muscle strength, ambulation capacity, and depression. Further well-designed studies are required to evaluate the long-term effects, cost-effectiveness, and satisfaction in larger samples.

## Introduction

Coronavirus disease 2019 (COVID-19), which is caused by severe acute respiratory syndrome coronavirus 2 (SARS-CoV-2), has resulted in unprecedented challenges for governments and healthcare workers worldwide since first identified at the end of 2019 (1, 2). As of April 30th, 2022, there have been more than 510 million confirmed cases related to COVID-19, including a shocking 6.2 million deaths (3). Clinical syndromes (e.g., dyspnea, hypoxia, and multiple organ failure) (4, 5), iatrogenic impairments (e.g., fatigue and muscle weakness) (6), and prolonged immobilization resulting from COVID-19 can significantly impact physical, psychological, and social functioning and reduce quality of life, which may persist for at least 6 months (7, 8). Apart from that, many survivors of COVID-19 have persistent symptoms and/or the development of long-term symptoms such as fatigue, headache, and dyspnea after infection, which is called as long COVID (9, 10). It is estimated that 5% of survivors of COVID-19 will need inpatient rehabilitation (11). Therefore, in addition to supportive therapy and medical treatment, rehabilitation plays an important role in COVID-19. However, given the fact that COVID-19 is a highly infectious disease and therefore healthcare facilities may be sources of contagion, new methods avoiding face-to-face contact between healthcare workers and patients are urgently needed.

Telerehabilitation is the provision of rehabilitation services to patients at a distance *via* information and communication technologies (12–14). Remote communication between patient and physical medicine or rehabilitation professional may occur through a number of technologies such as telephone (including text messaging), Internet, Internet-based videoconferencing, sensors (such as pedometers), or virtual reality programs (15, 16), in order to enable clinical rehabilitation services to be delivered to a satellite healthcare center or even directly to patients' homes (17). Telerehabilitation can provide physiotherapy, occupational therapy, speech therapy, telemonitoring, and teleconsultation without the physical presence of therapists or other healthcare workers (18). As technology advances and the availability of affordable devices and software increases, telerehabilitation may revolutionize the way in which rehabilitation is provided (19). Amidst the COVID-19 pandemic, the shortage of medical resources and isolation and quarantine measurements call for telerehabilitation services, as telerehabilitation offers an opportunity for homebound patients with COVID-19 to reach alternative rehabilitation services. Although there has been evidence supporting telerehabilitation for COVID-19 (20), low- to very low-quality evidence inevitably limits its conclusion and thus more meta-analyses are urgently needed. Additionally, there is still a lack of high-quality evaluation of the efficacy of telerehabilitation for quality of life, anxiety, and depression impacted by COVID-19. Recently, several randomized controlled trials (21–23) have been published and have yet to be reviewed. Consequently, a more comprehensive, rigorous, and high-quality meta-analysis of the current literature is important and desirable, which may further inform future research and implementation of telerehabilitation services.

As such, the aims of this meta-analysis were to analyze the randomized controlled trials published to data and to explore the efficacy of telerehabilitation for physical function, psychological function, and quality of life in patients with and survivors of COVID-19.

## Methods

The present meta-analysis followed the guidelines of Preferred Reporting Items for Systematic Review and Meta-analyses (Supplementary Material 1) (24). The protocol for this study was available on the International Prospective Register of Systematic Reviews platform (registration number: CRD42021297802). No ethical approval was needed as all information was extracted from studies published previously.

### Search strategy

We searched the Cochrane Library, EMBASE, MEDLINE (*via* PubMed), PEDro, ClinicalTrials.gov, and WHO International Clinical Trials Registry Platform (ICTRP) from January 1st,2020 to April 30th, 2022 using the following search terms: “COVID-19,” “SARS-CoV-2,” “telecommunications,” “randomized,” and other keywords confirmed following multiple pre-searches (Supplementary Material 2). In addition, we adjusted the terms according to the actual conditions to fit the requirements of each electronic database.

### Criteria for considering studies for this meta-analysis

The inclusion criteria were as follows: (1) Adults (age ≥18 years) with a current or past diagnosis of COVID-19; (2) The intervention should include telerehabilitation meeting the following definition: ”the delivery of rehabilitation services at a distance through communication and information technologies" (12–14), such as telephone (including text messaging), Internet, Internet-based videoconferencing, sensors (such as pedometers), or virtual reality programs (15, 16); (3) Tele-intervention should include at least 50% of rehabilitation being delivered remotely; (4) The comparison intervention was any face-to-face rehabilitation, usual care, or waiting without any therapy; (5) Studies have to evaluate at least one outcome about physical function, psychological function, or quality of life; (6) Only randomized controlled trials, which compared telerehabilitation with face-to-face rehabilitation or no rehabilitation or compared telerehabilitation plus usual care with usual care alone, were included; (7) The language of the articles was limited to English.

The exclusion criteria were as follows: (1) Studies in which the effect of telerehabilitation could not be separated from the effects of other therapies such as the telerehabilitation was combined with other therapies not included in the control group, which may affect the interpretation of the effects of telerehabilitation; (2) Studies utilizing a single treatment; (3) The comparison intervention was delivered remotely; (4) Review, editorial, and conference abstract, and non-English publications were excluded.

According to the above criteria, two investigators independently read the titles and abstracts of the retrieved records and eliminated obviously irrelevant studies, followed by a full-text retrieving of the remaining studies. Subsequently, two reviewers separately evaluated the articles for final inclusions. In case of ambiguity, we contacted the authors to provide additional information *via* email. All discrepancies were resolved through discussion, or by consulting a third investigator.

### Data extraction and quality assessment

Data on study title, first author information, year of publication, participants, experimental groups, control group(s), the protocol of intervention and control, follow-up, and the results of outcomes were extracted by two independent reviewers from included studies. The outcomes of interest were those related to physical function, psychological function, or quality of life. When quantitative data were not reported in text or supplementary materials, we extracted the data from figures using Engauge Digitizer 11.1 (25). The Engauge Digitizer is a tool that allows to recovers the data point from figures, which is the opposite of a graphing tool that converts data points to figures. Missing data items were requested from authors as the data has not been peer-reviewed.

Two investigators independently evaluated the risk of bias of included studies utilizing the Cochrane Collaboration's tool (26). High-bias risk, low-bias risk, and unclear bias risk were used to classify the included studies. In addition, these two reviewers separately summarized the overall quality of the evidence for key comparisons using the Grading of Recommendations Assessment, Development, and Evaluation (GRADE). Discrepancies were resolved through discussion, or by consulting a third investigator.

### Data analysis

All meta-analyses and graphical displays were conducted using Review Manager (RevMan) software (The Cochrane Collaboration, version 5.3). If methods of outcome measurement were different among the included studies, a standardized mean difference (SMD) was calculated; otherwise, a mean difference (MD) was used. To evaluate the statistical heterogeneity, we used the *I*^2^ statistic. If the value of *I*^2^ was < 50%, we utilize a fixed-effect model; otherwise, a random-effects model was utilized. To prevent double-counting sample sizes of control participants, we split the participant number of the control group in case of studies using a single control group and multiple experimental groups. We analyzed both absolute values and change from baseline when data were available. Where comparable data were available from at least two studies, we planned to conduct subgroup analyses in the following domains: disease status (COVID-19 patients vs. COVID-19 survivors), control design (conventional rehabilitation vs. no rehabilitation), underlying disease, and gender. We evaluated the robustness of the results using leave-one-out sensitivity analyses. The publication bias would not be analyzed using a funnel plot unless at least 10 studies were included in a certain subgroup. For studies that could not be included in the meta-analysis, we would perform a descriptive summary. Finally, to assess the type I error (false positive) produced by the cumulative meta-analysis, a trial sequential analysis (TSA) was conducted to confirm whether firm evidence was reached or not using TSA software (Copenhagen Trial Unit, Center for Clinical Intervention Research, Rigshospitalet, Copenhagen, Denmark, version 0.9.5.10 Beta) (27, 28).

## Results

### Results of the search

**Figure 1:**
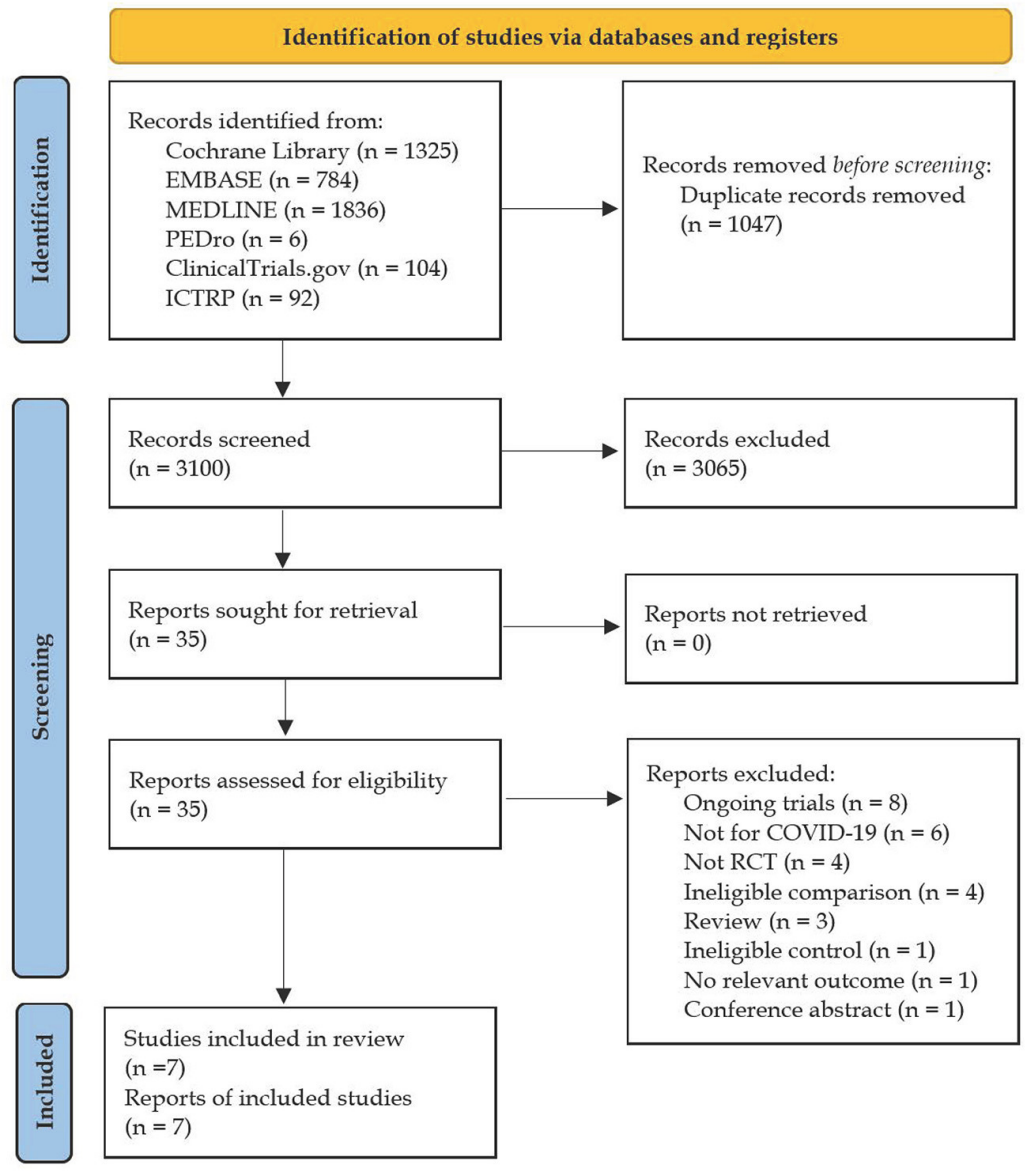
PRISMA flow diagram for search strategy and study selection.

Figure 1 shows the flow diagram for search strategy and study selection process. We initially retrieved 4,147 potentially eligible records and then we removed duplicates with 3,100 records left for title-abstract screening, resulting in 1,047 records being discarded, mostly because of irrelevant research topics. Thirty-five records were remained to determine their eligibility by carefully full-text screening. Subsequently, twenty-eight records were excluded from this review for various reasons. As a result, a total of seven studies (21–23, 29–32) were included and all of them were included in the quantitative synthesis.

### Characteristics of included studies

The characteristics of all studies included in this meta-analysis are provided in Table 1. Studies were published from 2020 to 2022. Of these, five studies (21, 22, 29–31) applied telerehabilitation to COVID-19 patients, while the remaining two (23, 32) investigated the efficacy of telerehabilitation for COVID-19 survivors. Variation in the telerehabilitation program was observed in the included studies. To improve the physical deconditioning and physiological deterioration, Rodriguez-Blanco et al. (29) employed a non-specific conditioning exercise program, which consisted of 10 exercises based on resistance and strength non-specific toning exercise. Gonzalez-Gerez et al. (30) delivered a respiratory rehabilitation remotely. Another study (31) utilized both breathing telerehabilitation and strength telerehabilitation. Li et al. (32) delivered an unsupervised 6-week home exercise program *via* a smartphone application called RehabApp, which offers breathing control, thoracic expansion, aerobic exercise, and strength exercise. Philip et al. (23) remotely delivered a 6-week online breathing and wellbeing program. Psychotherapy was delivered by the other two studies (21, 22). The intervention period ranged from 1 to 6 weeks. All studies assess the short-term effects of telerehabilitation, of which two studies (21, 32) also provided the result of follow-up for 28 weeks or 1 month.

**Table 1:** Characteristics of studies included in this meta-analysis, *K* = 7.

**References**	**Participants**	**Intervention**	**Control**	**Protocol**	**Outcome**	**Timing of measurement**	**Adverse events**
Rodriguez-Blanco et al. (29)	Patients with COVID-19 N: Intervention/Control = 18/18 Masculine gender: Intervention/Control = 9/10 Age (years), mean (SD): Intervention/Control = 39.0 (11.7)/41.3 (12.1) Comorbidities: not mentioned	Web-based Non-Specific Conditioning Exercise Program: 10 exercises based on non-specific toning exercises of resistance and strength	Wait without any therapy	Once a day for seven days, at patients' home	Borg scale; Six-min walking test; 30-s sit-to-stand test	Post-intervention	Not mentioned
Gonzalez-Gerez et al. (30)	Patients with COVID-19 N: Intervention/Control = 19/19 Masculine gender: Intervention/Control = 10/11 Age (years), mean (SD): Intervention/Control = 40.8 (9.8)/40.3 (12.5) Comorbidities: not mentioned	Web-based Breathing exercise program: 10 exercises based on the active cycle of breathing techniques	Wait without any therapy	Once a day for seven days, at patients' home	Borg scale; Multidimensional dyspnoea-12; Six-min walking test; 30-s sit-to-stand test	Post-intervention	Not mentioned
Rodríguez-Blanco et al. (31)	Patients with COVID-19 N: Intervention/Control = (29/26)/22 Masculine gender: Intervention/Control = (13/14)/10 Age (years), mean (SD): Intervention/Control = [41.9 (10.2)/34.8 (11.8)]/42.4 (11.8) Comorbidities: not mentioned	Group 1: Web-based Breathing exercise program: 10 exercises based on the active cycle of breathing techniques. Group 2: Web-based strength exercise program: 10 exercises based on strength exercises	Wait without any therapy	Once a day for 14 days, at patients' home	Borg scale; Multidimensional dyspnoea-12; Visual analog fatigue scale; Six-min walking test; 30-s sit-to-stand test	Post-intervention	Not mentioned
Li et al. (32)	Survivors of COVID-19 N: Intervention/Control = 52/60 Masculine gender: Intervention/Control= not mentioned Age (years), mean (SD): Intervention/Control=49.2 (10.8)/52.0 (11.1) Comorbidities: Intervention (3.4% heart disease, 13.6% hypertension, 13.6% diabetes, 15.3% obesity, 6.8% lung disease, and 27.1% other)/Control (11.7% heart disease, 30% hypertension, 15% diabetes, 13.33% obesity, 5% lung disease, and 20% other)	App-based exercise program: unsupervised breathing control and thoracic expansion, aerobic exercise, and lower limb muscle strength exercises specified in a three-tiered exercise plan with difficulty and intensity scheduled to increase over time	Short educational instructions at baseline	Three to four per week for 6 weeks, at patients' home	Six-min walking test; Squat time; Pulmonary function; Short Form Health Survey-12; Modified Medical Research Council	Post-intervention; 28 weeks after intervention	No serious adverse events were observed throughout the study period, except eight patients were hospitalized for non-life-threatening reasons unrelated to COVID-19 or telerehabilitation in the follow-up period
Philip et al. (23)	Survivors of COVID-19 N: Intervention/Control=58/71 Masculine gender: Intervention/Control = 14/12 Age (years), mean (SD): Intervention/Control = 49 (12)/50 (12) Comorbidities: The median (quartile) number of comorbidities was 1 (0-1) for the two groups	Online breathing and wellbeing program	Usual care	Weekly for 6 weeks	RAND 36-item short form; Chronic obstructive pulmonary disease assessment tool score; Visual analog scale for breathlessness; Dyspnea-12; Generalized anxiety disorder 7-item scale; Short form-6D	Post-intervention	No serious adverse events were observed, except one participant withdrew due to dizziness that they attributed to looking at the computer screen for too long
Wei et al. (22)	Patients with COVID-19 N: Intervention/Control = 13/13 Masculine gender: Intervention/Control = 9/7 Age (years), mean (SD): Intervention/Control = 40.8 (13.5)/48.5(9.5) Comorbidities: Intervention (38.5% any, 23.1% hypertension, 7.7% liver disease, and 7.7% heart disease)/Control (30.8% any, 7.7% hypertension, 7.7% liver disease, 7.7% gastric ulcer, and 7.7% acquired immune deficiency syndrome	Internet-based integrated program: a self-help intervention including breath relaxation training, mindfulness (body scan), “refuge” skills, and butterfly hug method	Daily supportive care	A fixed time every day for 2 weeks, at isolation ward	Hamilton Depression Rating Scale; Hamilton Anxiety Rating Scale	Post-intervention	Not mentioned
Liu et al. (21)	Patients with COVID-19 N: Intervention/Control = 126/126 Masculine gender: Intervention/Control = 70/80 Age (years), mean (SD): Intervention/Control = 43.8 (14.3)/41.5 (11.5) Comorbidities: not mentioned	Computerized cognitive behavioral therapy: relaxation mental imagery training, mindfulness meditation, and counting meditation	Treat as usual: periodic psychological assessments, general psychological support, and consultations discussing overall wellbeing and disease activity	Once a day for 1 week, at each trial center	Hamilton Depression Rating Scale; Hamilton Anxiety Rating Scale; Self-Rating Depression Scale; Self-Rating Anxiety Scale; Athens Insomnia Scale	Post-intervention; One month after intervention	Not mentioned

SD: standard deviation.

### Quality assessment

The risk of bias of the included studies is shown in Supplementary Material 3. High and unclear risks of bias were observed in the included studies. One study (22) did not report on the allocation concealment and thereby was classified as unclear risk for selection bias. Three studies (21, 23, 32) were classified as high risk for performance bias due to participants and personnel were not blinded, while another study (22) that did not explicitly report on this issue was rated as unclear risk of bias. Similarly, two studies (21, 22) were rated as high risk for detection bias due to the outcome assessor was not blinded. In addition, one study (30) did not report outcomes per the protocol and therefore was rated high risk for reporting bias. Based on the GRADE criteria, the quality of evidence was very low to moderate (Supplementary Material 4).

### The efficacy of telerehabilitation on dyspnea

**Figure 2:**
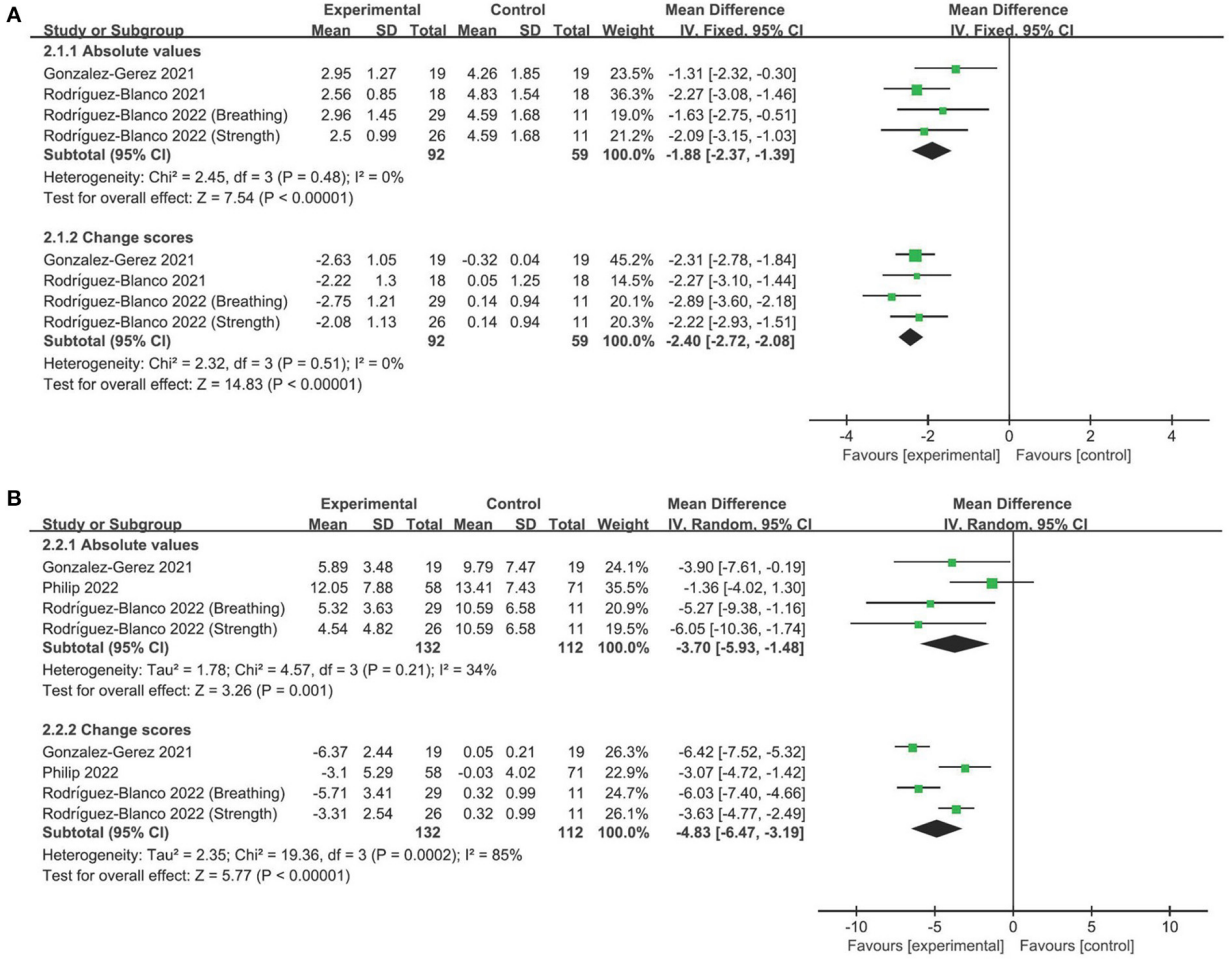
Forest plot analyses of the efficacy of telerehabilitation for **(A)** Borg scale and **(B)** Multidimensional dyspnea-12 questionnaire.

The Borg scale was utilized to assess dyspnea and presented results of both absolute values and change scores (29–31). There was moderate-quality evidence with a significant effect size favoring telerehabilitation relative to comparators (MD −1.88, 95% CI −2.37 to −1.39, *P* < 0.001; *I*^2^ = 0%; Figure 2A). The significant result persisted when change scores were used for analysis (MD −2.40, 95% CI −2.72 to −2.08, *P* < 0.001; *I*^2^ = 0%; Figure 2A). The overall finding persisted in the leave-one-out sensitivity analyses (Supplementary Material 5).

In addition, the Multidimensional dyspnea-12 questionnaire was used to evaluate the multidimensional nature of dyspnea. We found moderate-quality evidence that telerehabilitation significantly improved dyspnea relative to control groups. The finding did not differ between absolute value analysis (MD −3.70, 95% CI −5.93 to −1.48, *P* = 0.001; *I*^2^ = 34%; Figure 2B) and change score analysis (MD −4.83, 95% CI −6.47 to −3.19, *P* < 0.001; *I*^2^ = 85%; Figure 2B). Leave-one-out sensitivity analyses did not change the overall finding (Supplementary Material 5).

### The efficacy of telerehabilitation on lower limb strength

**Figure 3:**
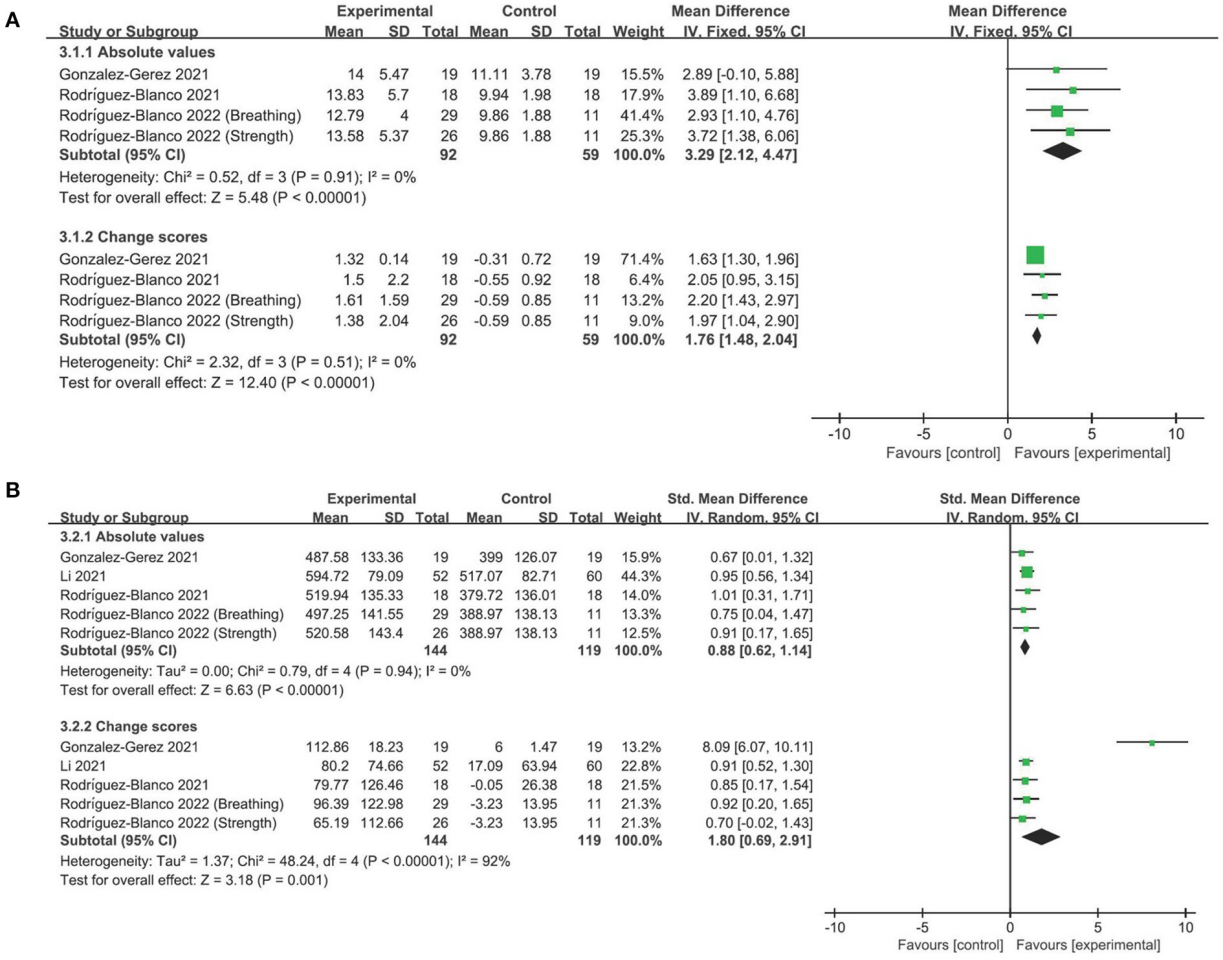
Forest plot analyses of the efficacy of telerehabilitation for **(A)** 30-s sit-to-stand test and **(B)** Six-min walking test.

To evaluate the performance of lower limbs, 30-s sit-to-stand test was used in three studies with four comparisons (29–31). We also found moderate-quality evidence supporting telerehabilitation, whether absolute values (MD 3.29, 95% CI 2.12–4.47, *P* < 0.001; *I*^2^ = 0%; Figure 3A) or change scores (MD 1.76, 95% CI 1.48–2.04, *P* < 0.001; *I*^2^ = 0%; Figure 3A) were employed. The overall finding did not differ after omitting any single study of the included studies (Supplementary Material 5).

### The efficacy of telerehabilitation on ambulation ability

For ambulation ability, studies utilizing the 6-min walking test exhibited moderate-quality evidence favoring telerehabilitation, irrespective of absolute values (SMD 0.88, 95% CI 0.62–1.14, *P* < 0.001; *I*^2^ = 0%; Figure 3B) or change values (SMD 1.80, 95% CI 0.69–2.91, *P* = 0.001; *I*^2^ = 92%; Figure 3B). Leave-one-out sensitivity analyses did not significantly change the overall finding (Supplementary Material 5).

### The efficacy of telerehabilitation on depression

**Figure 4:**
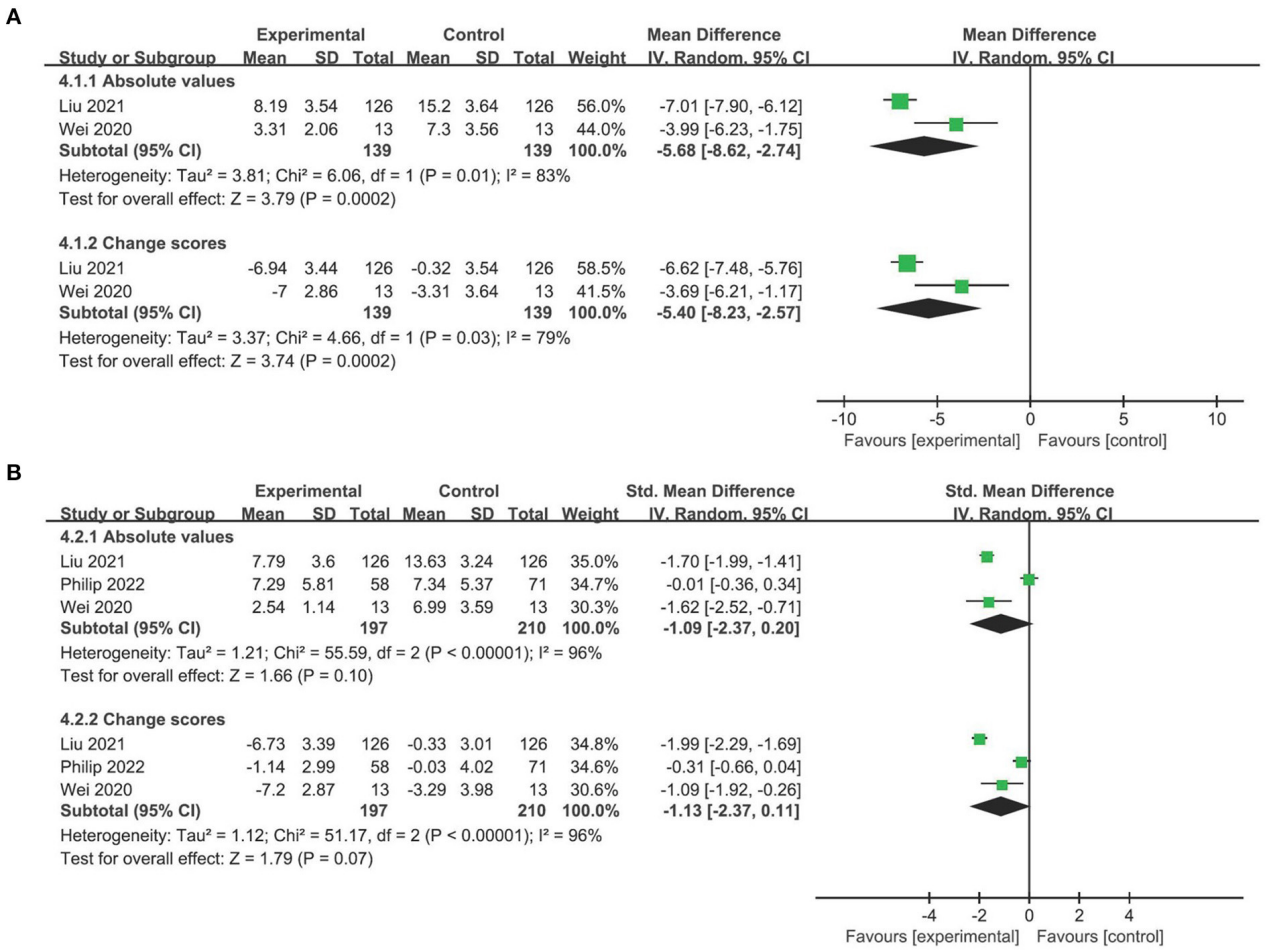
Forest plot analyses of the efficacy of telerehabilitation for **(A)** Hamilton depression rating scale and **(B)** Hamilton anxiety rating scale.

In terms of depression, studies employing the Hamilton depression rating scale exhibited very low-quality evidence supporting telerehabilitation, regardless of absolute values (MD −5.68, 95% CI −8.62 to −2.74, *P* < 0.001; *I*^2^ = 83%; Figure 4A) or change values (MD −5.40, 95% CI −8.23 to −2.57, *P* < 0.001; *I*^2^ = 79%; Figure 4A). The overall finding persisted in the leave-one-out sensitivity analyses (Supplementary Material 5).

### The efficacy of telerehabilitation on anxiety

No evidence found that telerehabilitation is superior over usual care, no matter absolute values (SMD −1.09, 95% CI −2.37 to 0.20, *P* = 0.10; *I*^2^ = 96%; Figure 4B) or change scores (SMD −1.13, 95% CI −2.37 to 0.11, *P* = 0.07; *I*^2^ = 96%; Figure 4B) were analyzed. Omitting the study by Philip et al. (23) rendered the result significantly (Supplementary Material 5).

### The efficacy of telerehabilitation on quality of life

For quality of life, no evidence favored the superiority of telerehabilitation, no matter absolute values (SMD 0.26, 95% CI −0.11 to 0.62, *P* = 0.16; *I*^2^ = 51%; Supplementary Material 6) or change scores (SMD 0.32, 95% CI −0.11 to 0.74, *P* = 0.14; *I*^2^ = 63%; Supplementary Material 6) were analyzed. Omitting the study by Philip et al. (23) rendered the result significantly (Supplementary Material 5).

### Trial sequential analysis

The TSA results indicated that firm evidence was reached for all positive outcomes, although the required information size was not met for all of them (Supplementary Material 7).

### Subgroup analysis and publication bias

The number and information of included studies were too insufficient to conduct reliable analyses of any predefined subgroup analysis and publication bias.

### Follow-up

Only one study (32) reported a follow-up assessment of the efficacy of telerehabilitation for physical function, which prevented further meta-analysis. Li et al. (32) reported that the efficacy of telerehabilitation for 6-min walking distance, lower limb muscle strength, and quality of life was maintained up to 28 weeks after intervention. Similarly, only one study (21) provided a follow-up assessment of telerehabilitation in psychological function and showed that the efficacy persisted 1 month after the intervention.

### Adverse events

Adverse events were reported by Li et al. (32), and they stated that no serious adverse events were observed throughout the study period, except eight patients were hospitalized for non-life-threatening reasons not related to COVID-19 or telerehabilitation in the follow-up period. Philip et al. (23) also reported no serious adverse events were observed, except one participant withdrew due to dizziness that they attributed to looking at the computer screen for too long. The other five studies (21, 22, 29–31) did not report on the occurrence of adverse events.

## Discussion

Overall, we found moderate- to low-quality evidence that telerehabilitation is effective and safe in the improvement of dyspnea, lower limb muscle strength, ambulation capacity, and depression. Of note, these results persisted in the leave-one-out sensitivity analyses, which may partly prove the robustness of the present meta-analysis. However, current evidence does not support the long-term effects of telerehabilitation for COVID-19. In addition, limited by the number and quality of included studies (five out of seven trials have some concerns; 71%) and the limited statistical inference, the aforementioned conclusions need to be verified through more high-quality studies. Therefore, further well-designed randomized controlled trials with large sample sizes for the short-term and long-term effects of telerehabilitation in the treatment of COVID-19 are needed. Further studies should particularly focus on patients who may benefit the most from telerehabilitation, but also should consider the technical requirement needed for reaching most homebound users, including, but not limited to cost-effectiveness, accessibility, and flexibility.

Telerehabilitation is a domain of telecommunications and telemedicine, which refers to a range of rehabilitation services that involve prevention, assessment, intervention, monitoring, supervision, education, counseling, and consultation (33). In this meta-analysis, we chose to focus on intervention and assessment of post-intervention, instead of telemonitoring alone because of the possibility of providing interventions, controlled by healthcare workers at a distance, with a rehabilitation aim (34). With these criteria, we systematically analyzed the efficacy of telerehabilitation for dysfunction affected by COVID-19, which might help inform future research direction and implementation of telerehabilitation. An important finding from this meta-analysis is that telerehabilitation is superior to no therapy or usual care for dyspnea, lower limb muscle strength, ambulation capacity, and depression. Facing the unprecedented pandemic, telerehabilitation has the potential to break the constraints of time and space and thus particularly help governments struggling to cope with the impact of the COVID-19 crisis.

Rehabilitation requires a sustained and coordinated effort from a multidisciplinary team, including the patient and his or her goals, family and friends, other caregivers (e.g., personal care attendants), physicians, nurses, physical and occupational therapists, speech-language pathologists, recreation therapists, psychologists, nutritionists, social workers, and others (35). It is to be noted that psychologists are recognized as important team members of rehabilitation in many rehabilitation guidelines (35, 36). As one kind of psychotherapy, cognitive behavioral therapy delivered in a hospital or at a distance was also recommended in rehabilitation guidelines (37) and systematic reviews (38, 39). Although psychotherapy such as cognitive behavioral therapy is often provided by psychologists, some physiotherapists also can provide it (40). Taken together, it is rational to classify psychotherapy provided at a distance into telerehabilitation and then include it in the present meta-analysis. Of the included studies, although psychotherapy was mainly provided by psychologists (21, 22), in the treatment of impaired psychological functioning affected by COVID-19, telerehabilitation is recommended to be delivered by a multidisciplinary team including physiotherapists, psychologists, nurses, physicians, etc., in future research and clinical practice.

Appropriate components comprised in the telerehabilitation programs are crucial factors that needed to be considered by designers or health professionals. In the present meta-analysis, we included studies with various telerehabilitation programs, including strength training, respiratory training, aerobic exercise, and psychotherapy. Of note, Li et al. (32) increased the intensity, duration, and difficulty of exercise during the experiment, which may ensure adequate exercise intensity and optimize the efficacy of telerehabilitation. However, other forms of telerehabilitation components are yet to be investigated. In addition, one study (41) reported that remote qigong exercise plus acupressure improved pulmonary function and cough in patients with severe COVID-19 and reduced hospital stay. However, we cannot separate the efficacy of remote qigong exercise from acupressure and therefore this study was excluded. As such, the telerehabilitation program that would best treat dysfunction affected by COVID-19 remains unclear.

Of the included studies, ambulation capacity assessed by the 6-min walking test is the most common outcome, followed by lower limb strength evaluated by the 30-s sit-to-stand test, dyspnea measured by the Borg scale and Multidimensional dyspnea-12 questionnaire, anxiety assessed by Hamilton anxiety rating scale and Generalized anxiety disorder 7-item scale, depression measured by Hamilton depression rating scale, and quality of life evaluated by Short Form Health Survey-12 and RAND 36-item short form. These assessment tools are valid and reliable methods to assess ambulation capacity (42), dyspnea (43, 44), lower limb muscle strength (45), depression (46), anxiety (47, 48), and quality of life (49, 50). It is suggested that the minimal clinically important differences are 0.9 (51) and 2.83 (52), respectively, for the Borg scale and Multidimensional dyspnea-12 questionnaire. And as suggested by the GRADE working group, the minimal clinically important difference can be considered as 0.5 when a standard mean difference was used for the 6-min walking test (53). Therefore, all of these outcomes reached minimal clinically important differences, which indicates these results are of clinical significance.

Gender and age are the factors to be taken into consideration in COVID-19 research. In terms of gender, one study shows that men with COVID-19 are prone to have worse outcomes and more death (54). And the result from another study indicated that men exhibited more robust inflammatory activation, which is evidenced by higher initial and peak inflammatory markers, as well as worse clinical outcomes (55). Taken together, men with COVID-19 are more likely to have worse clinical outcomes, thus compromising the efficacy of telerehabilitation. In the present meta-analysis, all the seven included studies recruited both male and female patients, and fortunately, all groups of the included studies were comparable at baseline. However, no stratified result was reported in the included studies, making the predefined subgroup analysis impossible. Regarding age, the highest death was observed in the oldest age group (age >70 years) relative to younger age groups (56), which may be associated with the generally high prevalence of comorbidities and weaker immune systems in order adults (57, 58). In the included studies, the mean age of patients with and survivors of COVID-19 ranged from 34.8 to 52.0. The reason why the included studies did not include older patients may due to the ability to use devices. Therefore, further generalization of the efficacy of telerehabilitation to older patients with COVID-19 needs further high-quality study with well-designed and easy-to-use devices. Apart from that, attention must be paid to educating healthcare workers to thoroughly understand the available telerehabilitation technologies and better encourage and instruct patients from all kinds of backgrounds to use the devices for telerehabilitation (59).

Comorbidity is also needed to be considered when conducting telerehabilitation studies for COVID-19. Studies found that hypertension and type 2 diabetes are the most common comorbidities, which may induce a more severe course of COVID-19 (56, 60, 61). Of the included studies, only three studies (22, 23, 32) measuring different outcomes reported comorbidities, which prevents further analysis of comorbidities. However, another study indicates that the efficacy of rehabilitation is not precluded by preexisting cardiorespiratory comorbidity in post-COVID-19 patients (62). As such, consideration must be given to further studies to explore whether the efficacy of telerehabilitation is varied by the preexisting comorbidities of participants.

Several indicators such as activities of daily living and quality of life play an important role in the assessment of the efficacy of telerehabilitation for COVID-19. It is reported that approximately half of post-COVID-19 patients had low physical functioning and impaired performance of activities of daily living (63). However, quality of life was only assessed by two studies (23, 32) with contradictory conclusions, and activities of daily living (such as Barthel Index score) were not evaluated in any of the included studies. Additionally, physical activity measured by Rapid Assessment of Physical Activity, Short Recall Physical Activity Questionnaires, short physical performance battery, or others is another outcome that must be taken into consideration in future studies. Further research into these domains is therefore warranted to comprehensively evaluate the efficacy of telerehabilitation for patients with COVID-19. In addition, patient satisfaction in telerehabilitation intervention was not investigated in the included studies, which may inevitably affect the promotion of telerehabilitation.

## Strengths and limitations

The strength of this meta-analysis was that we included the most relevant randomized controlled trials based on the most rigorous criteria for inclusion and exclusion, which is a useful tool for telerehabilitation decision-making and program planning and helps to identify areas in which study is still scarce (64). In addition, to obtain the most reasonable results and thereby inform future research direction and implementation of telerehabilitation, we utilized the Cochrane Collaboration's tool to assess the risk of bias of each included study and the GRADE tool to evaluate the overall evidence quality of key outcomes, which increase the confidence in our findings (65). Finally, the TSA was provided to assess whether firm evidence was reached or not, and the results indicated that some findings were robust enough to deserve prompt clinical consideration in routine clinical practice. However, TSA for Multidimensional dyspnea-12 questionnaire, 6-min walking test, and Hamilton depression rating scale showed current sample size did not reach the required information size with more additional studies needed.

Notwithstanding its significant findings, several limitations inevitably existed in this meta-analysis. A limitation is that there might have been several significant heterogeneities in the included studies, such as training program, duration, and disease status, which might have caused uncontrolled bias in the meta-analyses. Another limitation lay in the small sample sizes (three of seven trials have very small sample sizes), which might have affected the statistical power of this meta-analysis. The reason why the demographics of some studies are not addressed at the scale one would expect can be explained by the fact that COVID-19 is a relatively novel and unprecedented disease and therefore the implementation of telerehabilitation with patients with COVID-19 is a new and understandably scarcely diffused approach (34, 59). When evaluating the overall evidence quality of key outcomes using the GRADE tool, the evidence was downgraded one level if the total sample size was < 400 (as a rule of thumb for implementing GRADE 'optimal information size' criteria) (66). Further study is, therefore, warranted to verify and strengthen the current conclusions. In addition, the small number of included randomized controlled trials prevented further analysis, leaving some unsolved knowledge gaps such as the long-term effects of telerehabilitation and people who might benefit from telerehabilitation. Furthermore, it is thought that telerehabilitation may result in cost savings, however, the cost-effectiveness was not reported in any studies included. Although Hamilton scales and Generalized anxiety disorder 7-item scale are the most commonly used outcome in clinical and research practice, and the literature supports their reliability, validity, and sensitivity (46, 48, 67), they inevitably have recall bias and are still not an easy task to measure these conditions. However, one has very few options besides the use of these validated questionnaires in the era of COVID-19. More objective and comprehensive measurement methods for depression and anxiety impacted by COVID-19 should be developed and adopted in the future. Apart from that, four included studies were conducted in Europe, while the other three were in China. Therefore, there is a need to promote and implement telerehabilitation in regions other than Europe and China, taking advantage of policy support such as coverage of cost by government or medical insurance (39). Of the included studies, three (29–31) were conducted by the same research team. It is therefore possible that the group undertaking the studies has excellent expertise, which may enhance the efficacy of telerehabilitation. More research groups are encouraged to participate in telerehabilitation to confirm its effectiveness, as previous studies and the present meta-analysis suggest telerehabilitation is a promising strategy to treat patients with and survivors of COVID-19. In the end, although the most mainstream electronic databases and clinical trials registry platforms were retrieved for this meta-analysis, the language of the included trials was confined to English, leaving studies reported in other languages might not to be included.

## Implications for future research

Future studies should therefore optimize the experimental design, such as providing the sample size calculation, expanding the number of participants, and conducting in a double-blind fashion, to increase our confidence in estimating the effects of telerehabilitation. In addition, long-term follow-up, cost-effectiveness, satisfaction, and the profile of telerehabilitation users are factors that must be taken into consideration in future studies. COVID-19 calls for heavy demand for rehabilitation services and thus induces a heavy economic burden. If telerehabilitation, however, could have comparable effectiveness to face-to-face rehabilitation but in a more cost-effective manner (such as significantly reducing the burden of travel), this strategy could be further promoted as a viable alternative to deliver rehabilitation services during and even after the COVID-19 pandemic (59). Apart from that, we strongly recommend future studies be conducted per the CONSORT guideline and accurately report all relevant outcomes (including but not limited to physical, psychological, and social functioning, activities of daily living, and quality of life) in the forms of absolute values and change scores and if possible, provide stratified results in terms of the severity of disease, age, gender, and comorbidities, which may facilitate future systematic reviews and thus allow more robust and precise findings.

## Conclusions

Telerehabilitation may be an effective and safe option for improving patients with and survivors of COVID-19 in dyspnea, lower limb muscle strength, ambulation capacity, and depression. Caution must be taken when interpreting these findings since the current evidence is limited by the number and quality of included studies and the limited statistical inference. Further well-designed studies are required to evaluate the long-term effects, cost-effectiveness, and satisfaction in larger samples, as well as to pay more attention to patients with COVID-19 who may benefit the most from telerehabilitation.

## Data availability statement

The original contributions presented in the study are included in the article/supplementary material, further inquiries can be directed to the corresponding author/s.

## Author contributions

JH and YF conceived and designed the study. JH, YF, and CY developed the search strategy. JH, ZZ, and TW screened abstracts and full-text reports. JH, YF, and YC extracted outcomes. JY and KZ the interpretation of the data. JH and YQ wrote the manuscript. All authors contributed to the article and approved the submitted version.

## Funding

The research reported in this publication was supported by the National Key R&D Plan (2017YFC1308504 and 2017YFC1308500), National Natural Science Foundation (81902287), Project of Science & Technology Department of Sichuan Province (2021YJ0184), and Scientific Research Project of Health Commission of Sichuan Province (20PJ035).

## Conflict of interest

The authors declare that the research was conducted in the absence of any commercial or financial relationships that could be construed as a potential conflict of interest.

## Publisher's note

All claims expressed in this article are solely those of the authors and do not necessarily represent those of their affiliated organizations, or those of the publisher, the editors and the reviewers. Any product that may be evaluated in this article, or claim that may be made by its manufacturer, is not guaranteed or endorsed by the publisher.
