# Supplementary material for: Do patients with and survivors of COVID-19 benefit from telerehabilitation? A meta-analysis of randomized controlled trials
Source: Front Public Health. 2022 Sep 28;10:954754. doi: 10.3389/fpubh.2022.954754 (PMC9555811; doi:10.3389/fpubh.2022.954754)

Supplementary Material

**Supplementary Material 1:** PRISMA checklist.

**Supplementary Material 2:** Literature search strategy used in the current review.

**Supplementary Material 3:** Risk of bias graph summary of the included studies.

**Supplementary Material 4:** GRADE Profile of the Included Studies.

**Supplementary Material 5:** Results of leave-one-out sensitivity analysis investigating the efficacy of Telerehabilitation for COVID-19.

**Supplementary Material 6:** Forest plot analyses of the efficacy of telerehabilitation for quality of life.

**Supplementary Material 7:** Trials sequential analysis for outcomes of interest.

**Supplementary Material 1.** PRISMA checklist.

| **Section and Topic** | **Item #** | **Checklist item** | **Location where item is reported** |
| --- | --- | --- | --- |
| **TITLE** | | |  |
| Title | 1 | Identify the report as a systematic review. | Page 1 |
| **ABSTRACT** | | |  |
| Abstract | 2 | See the PRISMA 2020 for Abstracts checklist. | Page 1-2 |
| **INTRODUCTION** | | |  |
| Rationale | 3 | Describe the rationale for the review in the context of existing knowledge. | Page 2-3 |
| Objectives | 4 | Provide an explicit statement of the objective(s) or question(s) the review addresses. | Page 3 |
| **METHODS** | | |  |
| Eligibility criteria | 5 | Specify the inclusion and exclusion criteria for the review and how studies were grouped for the syntheses. | Page 3-4 |
| Information sources | 6 | Specify all databases, registers, websites, organisations, reference lists and other sources searched or consulted to identify studies. Specify the date when each source was last searched or consulted. | Page 3 |
| Search strategy | 7 | Present the full search strategies for all databases, registers and websites, including any filters and limits used. | Supplement 2 |
| Selection process | 8 | Specify the methods used to decide whether a study met the inclusion criteria of the review, including how many reviewers screened each record and each report retrieved, whether they worked independently, and if applicable, details of automation tools used in the process. | Page 4 |
| Data collection process | 9 | Specify the methods used to collect data from reports, including how many reviewers collected data from each report, whether they worked independently, any processes for obtaining or confirming data from study investigators, and if applicable, details of automation tools used in the process. | Page 4 |
| Data items | 10a | List and define all outcomes for which data were sought. Specify whether all results that were compatible with each outcome domain in each study were sought (e.g. for all measures, time points, analyses), and if not, the methods used to decide which results to collect. | Page 4 |
|  | 10b | List and define all other variables for which data were sought (e.g. participant and intervention characteristics, funding sources). Describe any assumptions made about any missing or unclear information. | Page 4 |
| Study risk of bias assessment | 11 | Specify the methods used to assess risk of bias in the included studies, including details of the tool(s) used, how many reviewers assessed each study and whether they worked independently, and if applicable, details of automation tools used in the process. | Page 4 |
| Effect measures | 12 | Specify for each outcome the effect measure(s) (e.g. risk ratio, mean difference) used in the synthesis or presentation of results. | Page 4 |
| Synthesis methods | 13a | Describe the processes used to decide which studies were eligible for each synthesis (e.g. tabulating the study intervention characteristics and comparing against the planned groups for each synthesis (item #5)). | Page 4 |
|  | 13b | Describe any methods required to prepare the data for presentation or synthesis, such as handling of missing summary statistics, or data conversions. | Page 4 |
|  | 13c | Describe any methods used to tabulate or visually display results of individual studies and syntheses. | Page 4 |
|  | 13d | Describe any methods used to synthesize results and provide a rationale for the choice(s). If meta-analysis was performed, describe the model(s), method(s) to identify the presence and extent of statistical heterogeneity, and software package(s) used. | Page 4 |
|  | 13e | Describe any methods used to explore possible causes of heterogeneity among study results (e.g. subgroup analysis, meta-regression). | Page 4 |
|  | 13f | Describe any sensitivity analyses conducted to assess robustness of the synthesized results. | Page 4 |
| Reporting bias assessment | 14 | Describe any methods used to assess risk of bias due to missing results in a synthesis (arising from reporting biases). | Page 3 |
| Certainty assessment | 15 | Describe any methods used to assess certainty (or confidence) in the body of evidence for an outcome. | Page 3 |
| **RESULTS** | | |  |
| Study selection | 16a | Describe the results of the search and selection process, from the number of records identified in the search to the number of studies included in the review, ideally using a flow diagram. | Page 4-5  Figure 1 |
|  | 16b | Cite studies that might appear to meet the inclusion criteria, but which were excluded, and explain why they were excluded. | Figure 1 |
| Study characteristics | 17 | Cite each included study and present its characteristics. | Page 5  Table 1 |
| Risk of bias in studies | 18 | Present assessments of risk of bias for each included study. | Page 5  Supplement 3 |
| Results of individual studies | 19 | For all outcomes, present, for each study: (a) summary statistics for each group (where appropriate) and (b) an effect estimate and its precision (e.g. confidence/credible interval), ideally using structured tables or plots. | Page 5-6  Figure 2-4 |
| Results of syntheses | 20a | For each synthesis, briefly summarise the characteristics and risk of bias among contributing studies. | Page 5-6  Table 1 |
|  | 20b | Present results of all statistical syntheses conducted. If meta-analysis was done, present for each the summary estimate and its precision (e.g. confidence/credible interval) and measures of statistical heterogeneity. If comparing groups, describe the direction of the effect. | Page 5-6  Figure 2-4 |
|  | 20c | Present results of all investigations of possible causes of heterogeneity among study results. | Figure 2-4  Supplement 4 |
|  | 20d | Present results of all sensitivity analyses conducted to assess the robustness of the synthesized results. | Page 5-6  Supplementl 5 |
| Reporting biases | 21 | Present assessments of risk of bias due to missing results (arising from reporting biases) for each synthesis assessed. | Page 5  Supplement 3 |
| Certainty of evidence | 22 | Present assessments of certainty (or confidence) in the body of evidence for each outcome assessed. | Page 5  Supplement 4 |
| **DISCUSSION** | | |  |
| Discussion | 23a | Provide a general interpretation of the results in the context of other evidence. | Page 7 |
|  | 23b | Discuss any limitations of the evidence included in the review. | Page 9 |
|  | 23c | Discuss any limitations of the review processes used. | Page 9 |
|  | 23d | Discuss implications of the results for practice, policy, and future research. | Page 10 |
| **OTHER INFORMATION** | | |  |
| Registration and protocol | 24a | Provide registration information for the review, including register name and registration number, or state that the review was not registered. | Page 3 |
|  | 24b | Indicate where the review protocol can be accessed, or state that a protocol was not prepared. | Page 3 |
|  | 24c | Describe and explain any amendments to information provided at registration or in the protocol. | N/A |
| Support | 25 | Describe sources of financial or non-financial support for the review, and the role of the funders or sponsors in the review. | Page 10 |
| Competing interests | 26 | Declare any competing interests of review authors. | Page 10 |
| Availability of data, code and other materials | 27 | Report which of the following are publicly available and where they can be found: template data collection forms; data extracted from included studies; data used for all analyses; analytic code; any other materials used in the review. | Page 3 |

**Supplementary Material 2.** Literature search strategy used in the current review.

1. Database: Cochrane Library (from January 1st, 2020 to April 30th, 2022)

- #1 MeSH descriptor: [COVID-19] explode all trees
- # 2 MeSH descriptor: [SARS-CoV-2] explode all trees
- # 3 #1 OR #2
- # 4 Coronavirus Disease 2019 or Coronavirus Disease 19 or Coronavirus Disease-19 or Severe Acute Respiratory Syndrome Coronavirus 2 Infection or covid 19 or covid-19 or SARS-COV-2 or 2019 nCoV Disease* or 2019 nCoV infection*
- # 5 #3 OR #4
- # 6 MeSH descriptor: [Telecommunications] explode all trees
- # 7 telecommunication* or teleconference* or tele-conference* or telemediicine or tele-medicine or telemetry or telerehab* or tele-rehab* or telehealth or tele-health or telehomecare or tele-homecare or telecoach or tele-coach or videoconference* or video-conferenc* or videoconsult* or video-consultat* or teleconsultat* or tele-consultat* or telecare* or tele-care* or teletherap* or tele-therap* or telecounsel* or tele-counsel* or Remote Consultation or remote rehabilitation* or virtual rehabilitation* or ehealth or e-health or eMedicine or e-Medicine or eRehab* or eRehab* or "mobile health" or mhealth or m-health or information technolog* OR information communication technolog* OR ICT or APP or web OR internet* OR wireless* OR wireless*
- # 8 #6 OR #7
- #9 Random or Random Allocation or Randomized controlled trials
- #10 #5 AND #8 AND #9

1. Database: EMBASE (from January 1st, 2020 to April 30th, 2022)

- #1 exp COVID 19/
- #2 exp SARS-CoV-2/
- #3 1 or 2
- #4 (Coronavirus Disease 2019 or Coronavirus Disease 19 or Coronavirus Disease-19 or Severe Acute Respiratory Syndrome Coronavirus 2 Infection or covid 19 or covid-19 or SARS-COV-2 or 2019 nCoV Disease* or 2019 nCoV infection*).af.
- #5 3 or 4
- #6 exp Telecommunications/
- #7 (telecommunication* or teleconference* or tele-conference* or telemediicine or tele-medicine or telemetry or telerehab* or tele-rehab* or telehealth or tele-health or telehomecare or tele-homecare or telecoach or tele-coach or videoconference* or video-conferenc* or videoconsult* or video-consultat* or teleconsultat* or tele-consultat* or telecare* or tele-care* or teletherap* or tele-therap* or telecounsel* or tele-counsel* or Remote Consultation or remote rehabilitation* or virtual rehabilitation* or ehealth or e-health or eMedicine or e-Medicine or eRehab* or eRehab* or "mobile health" or mhealth or m-health or information technolog* or information communication technolog* or ICT or APP or web or internet* or wireless* or wireless*).af.
- #8 6 or 7
- #9 (Random or Random Allocation or Randomized controlled trials).af.
- #9 5 and 8 and 9

1. Database: MEDLINE (from January 1st, 2020 to April 30th, 2022)

- #1 (COVID 19 or SARS-CoV-2[MeSH Terms]) OR (Coronavirus Disease 2019 or Coronavirus Disease 19 or Coronavirus Disease-19 or Severe Acute Respiratory Syndrome Coronavirus 2 Infection or covid 19 or covid-19 or SARS-COV-2 or 2019 nCoV Disease* or 2019 nCoV infection*)
- #2 (telecommunication*[MeSH Terms]) OR (telecommunication* or teleconference* or tele-conference* or telemediicine or tele-medicine or telemetry or telerehab* or tele-rehab* or telehealth or tele-health or telehomecare or tele-homecare or telecoach or tele-coach or videoconference* or video-conferenc* or videoconsult* or video-consultat* or teleconsultat* or tele-consultat* or telecare* or tele-care* or teletherap* or tele-therap* or telecounsel* or tele-counsel* or Remote Consultation or remote rehabilitation* or virtual rehabilitation* or ehealth or e-health or eMedicine or e-Medicine or eRehab* or eRehab* or "mobile health" or mhealth or m-health or information technolog* OR information communication technolog* OR ICT or APP or web OR internet* OR wireless* OR wireless*)
- #3 random*
- #4 #1 AND #2 AND #3

1. Database: PEDro (from January 1st, 2020 to April 30th, 2022)

- tele (in asbtract or title) AND clinical trial (method) AND cardiothoracis (subdiscipline)

1. ClinicalTrials.gov (from January 1st, 2020 to April 30th, 2022)

- Interventional Studies | COVID-19 | telemedicine OR telerehabilitation | Start date on or after 01/01/2020

1. WHO International Clinical Trials Registry Platform (ICTRP; from January 1st, 2020 to April 30th, 2022)

- COVID-19 AND telemedicine OR COVID-19 AND telerehabilitation


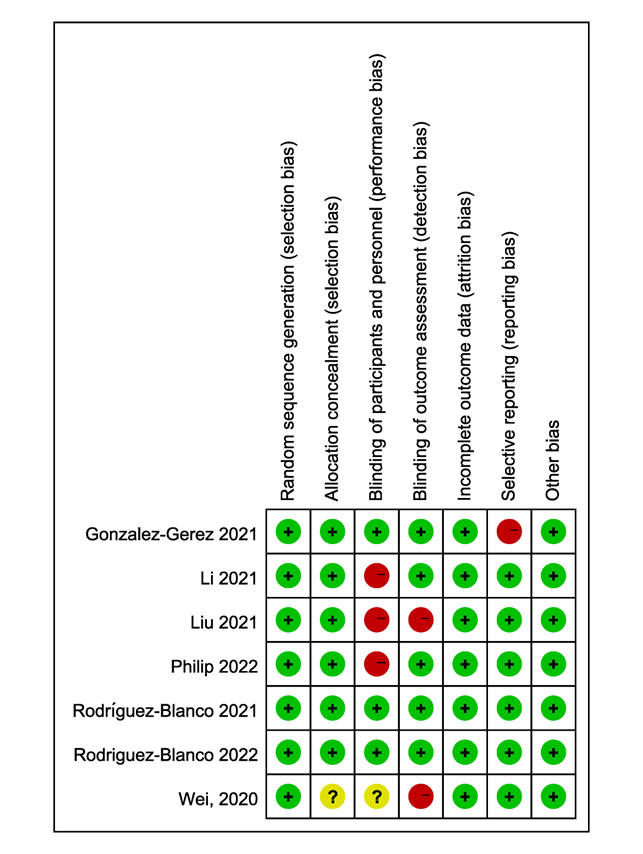


**Supplementary Material 3**. Risk of bias graph summary of the included studies.

**Supplementary Material 4.** GRADE Profile of the Included Studies.

| **Certainty Assessment** | | | | | | | **No. of Patients** | | **Certainty** | **Importance** |
| --- | --- | --- | --- | --- | --- | --- | --- | --- | --- | --- |
| **No. of Studies** | **Study Design** | **Risk of Bias** | **Inconsistency** | **Indirectness** | **Imprecision** | **Other Considerations** | **Telerehabilitation** | **control** |  |  |
| Borg scale | | | | | | | | | | |
| 3 | randomised trials | not serious | not serious | not serious | serious ^a^ | none | 92 | 59 | ⨁⨁⨁◯ MODERATE | CRITICAL |
| Multidimensional dyspnoea-12 | | | | | | | | | | |
| 3 | randomised trials | not serious | not serious | not serious | serious ^a^ | none | 132 | 112 | ⨁⨁⨁◯ MODERATE | CRITICAL |
| 30-seconds sit-to-stand test | | | | | | | | | | |
| 3 | randomised trials | not serious | not serious | not serious | serious ^a^ | none | 92 | 59 | ⨁⨁⨁◯ MODERATE | CRITICAL |
| Six-minute walking test | | | | | | | | | | |
| 4 | randomised trials | not serious | seriousc | not serious | serious ^a^ | none | 144 | 119 | ⨁⨁⨁◯ MODERATE | CRITICAL |
| Hamilton depression rating scale | | | | | | | | | | |
| 2 | randomised trials | Serious ^b^ | serious^c^ | not serious | serious ^a^ | not serious | 139 | 139 | ⨁◯◯◯ VERY LOW | IMPORTANT |
| Anxiety | | | | | | | | | | |
| 3 | randomised trials | Serious ^b^ | serious^c^ | not serious | not serious | not serious | 197 | 210 | ⨁⨁◯◯ LOW | IMPORTANT |
| Quality of life | | | | | | | | | | |
| 2 | randomised trials | Serious ^b^ | not serious | not serious | serious ^a^ | not serious | 110 | 131 | ⨁⨁◯◯ LOW | CRITICAL |

^a^ Downgraded one level due to total sample size being less than 400 (as a rule of thumb for implementing GRADE 'optimal information size' criteria).

^b^ Downgraded one level due to serious risk of bias (dominated by unclear and high risk of bias).

^b^ Downgraded one level due to high heterogeneity.

**Supplementary Material 5.** Results of leave-one-out sensitivity analysis investigating the efficacy of telerehabilitation for COVID-19.

| Variables | Forms | Study excluded | Overall analysis | | |
| --- | --- | --- | --- | --- | --- |
|  |  |  | MD/SMD | *I*^2^ (%) | *p* |
| Borg scale | Absolute values | Gonzalez-Gerez et al. 2021 | -2.06 [-2.62, -1.50] | 0 | <.001 |
|  |  | Rodríguez-Blanco et al. 2021 | -1.66 [-2.28, -1.05] | 0 | <.001 |
|  |  | Rodríguez-Blanco et al. 2022 (Breathing) | -1.94 [-2.49, -1.40] | 9 | <.001 |
|  |  | Rodríguez-Blanco et al. 2022 (Strength) | -1.83[-2.38, -1.28] | 12 | <.001 |
|  |  |  |  |  |  |
|  | Change scores | Gonzalez-Gerez et al. 2021 | -2.47 [-2.90, -2.05] | 3 | <.001 |
|  |  | Rodríguez-Blanco et al. 2021 | -2.42 [-2.77, -2.08] | 9 | <.001 |
|  |  | Rodríguez-Blanco et al. 2022 (Breathing) | -2.28 [-2.63, -1.92] | 0 | <.001 |
|  |  | Rodríguez-Blanco et al. 2022 (Strength) | -2.45 [-2.80, -2.09] | 0 | <.001 |
|  |  |  |  |  |  |
| Multidimensional dyspnea-12 questionnaire | Absolute values | Gonzalez-Gerez et al. 2021 | -3.85 [-7.00, -0.70] | 55 | 0.02 |
|  |  | Rodríguez-Blanco et al. 2022 (Breathing) | -3.36 [-6.07, -0.65] | 45 | 0.02 |
|  |  | Rodríguez-Blanco et al. 2022 (Strength) | -3.09 [-5.44, -0.74] | 30 | 0.01 |
|  |  | Philip et al. 2022 | -4.96 [-7.28, -2.64] | 0 | <.001 |
|  |  |  |  |  |  |
|  | Change scores | Gonzalez-Gerez et al. 2021 | -4.26 [-6.00, -2.53] | 79 | <.001 |
|  |  | Rodríguez-Blanco et al. 2022 (Breathing) | -6.27 [-6.53, -2.31] | 88 | <.001 |
|  |  | Rodríguez-Blanco et al. 2022 (Strength) | -5.25 [-7.14, -3.37] | 83 | <.001 |
|  |  | Philip et al. 2022 | -5.35 [-7.14, -3.56] | 85 | <.001 |
|  |  |  |  |  |  |
| 30-seconds sit-to-stand test | Absolute values | Gonzalez-Gerez et al. 2021 | 3.37 [2.09, 1.65] | 0 | <.001 |
|  |  | Rodríguez-Blanco et al. 2021 | 3.17 [1.87, 4.46] | 0 | <.001 |
|  |  | Rodríguez-Blanco et al. 2022 (Breathing) | 3.55 [2.01, 5.09] | 0 | <.001 |
|  |  | Rodríguez-Blanco et al. 2022 (Strength) | 3.15 [1.79, 4.51] | 0 | <.001 |
|  |  |  |  |  |  |
|  | Change scores | Gonzalez-Gerez et al. 2021 | 2.09 [1.57, 2.62] | 0 | <.001 |
|  |  | Rodríguez-Blanco et al. 2021 | 1.74 [1.46, 2.03] | 2 | <.001 |
|  |  | Rodríguez-Blanco et al. 2022 (Breathing) | 1.70 [1.40, 2.00] | 0 | <.001 |
|  |  | Rodríguez-Blanco et al. 2022 (Strength) | 1.74 [1.45, 2.03] | 5 | <.001 |
|  |  |  |  |  |  |
| Six-minute walking test ^a^ | Absolute values | Gonzalez-Gerez et al. 2021 | 0.92 [0.64, 1.21] | 0 | <.001 |
|  |  | Li et al. 2021 | 0.83 [0.48, 1.18] | 0 | <.001 |
|  |  | Rodríguez-Blanco et al. 2021 | 0.86 [0.58, 1.14] | 0 | <.001 |
|  |  | Rodríguez-Blanco et al. 2022 (Breathing) | 0.90 [0.62, 1.18] | 0 | <.001 |
|  |  | Rodríguez-Blanco et al. 2022 (Strength) | 0.88 [0.60, 1.16] | 0 | <.001 |
|  |  |  |  |  |  |
|  | Change scores | Gonzalez-Gerez et al. 2021 | 0.87 [0.59, 1.15] | 0 | <.001 |
|  |  | Li et al. 2021 | 2.29 [0.57, 4.00] | 94 | 0.009 |
|  |  | Rodríguez-Blanco et al. 2021 | 2.19 [0.71, 3.67] | 94 | 0.004 |
|  |  | Rodríguez-Blanco et al. 2022 (Breathing) | 2.16 [0.71, 3.61] | 94 | 0.004 |
|  |  | Rodríguez-Blanco et al. 2022 (Strength) | 2.21 [0.77, 3.66] | 94 | 0.003 |
|  |  |  |  |  |  |
| Hamilton depression rating scale | Absolute values | Liu et al. 2021 | -3.99 [-6.23, -1.75] | - | 0.001 |
|  |  | Wei et al. 2020 | -7.01 [-7.90, -6.12] | - | <.001 |
|  |  |  |  |  |  |
|  | Change scores | Liu et al. 2021 | -3.69 [-6.21, -1.17] | - | 0.004 |
|  |  | Wei et al. 2020 | -6.62 [-7.48, -5.76] | - | <.001 |
|  |  |  |  |  |  |
| Anxiety ^a^ | Absolute values | Liu et al. 2021 | -0.76 [-2.33, 0.82] | 91 | 0.35 |
|  |  | Philip et al. 2022 | -1.69 [-1.97, -1.42] | 0 | <.001 |
|  |  | Wei et al. 2020 | -0.86 [-2.51, 0.80] | 98 | 0.31 |
|  |  |  |  |  |  |
|  | Change scores | Liu et al. 2021 | -0.60 [-1.35, 0.14] | - | 0.004 |
|  |  | Philip et al. 2022 | -1.63 [-2.49, -0.76] | 75 | 0.001 |
|  |  | Wei et al. 2020 | -1.15 [-2.80, 0.50] | 98 | 0.17 |
|  |  |  |  |  |  |
| Quality of life ^a^ | Absolute values | Li et al. 2021 | 0.08 [-0.27, 0.43] | - | 0.65 |
|  |  | Philip et al. 2022 | 0.45 [0.08, 0.83] | - | 0.02 |
|  |  |  |  |  |  |
|  | Change scores | Li et al. 2021 | 0.11 [-0.24, 0.45] | - | 0.55 |
|  |  | Philip et al. 2022 | 0.54 [0.16, 0.92] | - | 0.005 |


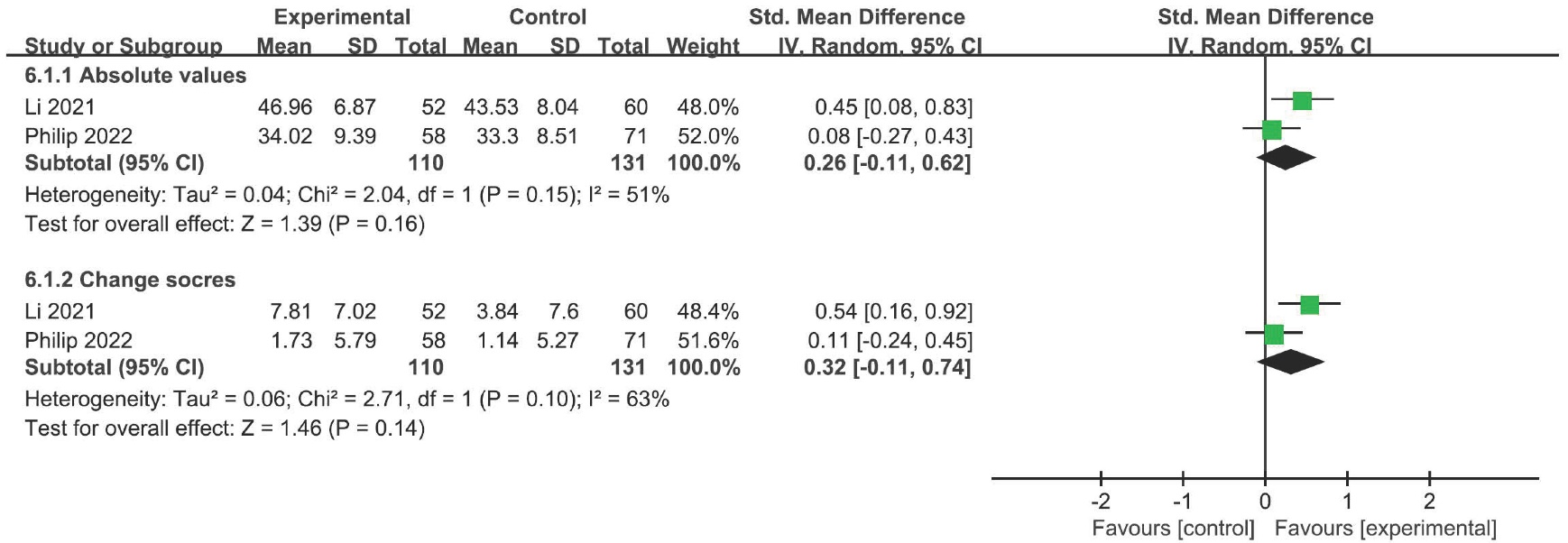


**Supplementary Material 6:** Forest plot analyses of the efficacy of telerehabilitation for quality of life.

**Supplementary Material 7:** Trials sequential analysis for outcomes of interest.

1. Trials sequential analysis for outcomes of interest.
2. Supplementary Material 7A. Trial sequential analysis for Borg scale. Uppermost and lowermost curves represent trial sequential monitoring boundary lines (red) for benefit and harm, respectively. Horizontal lines (brown) represent the traditional boundaries for statistical significance. The cumulative Z curve (blue) crosses the monitoring boundary curve (red), indicating firm evidence that telerehabilitation is superior to no therapy; meanwhile, it reached the required information size.


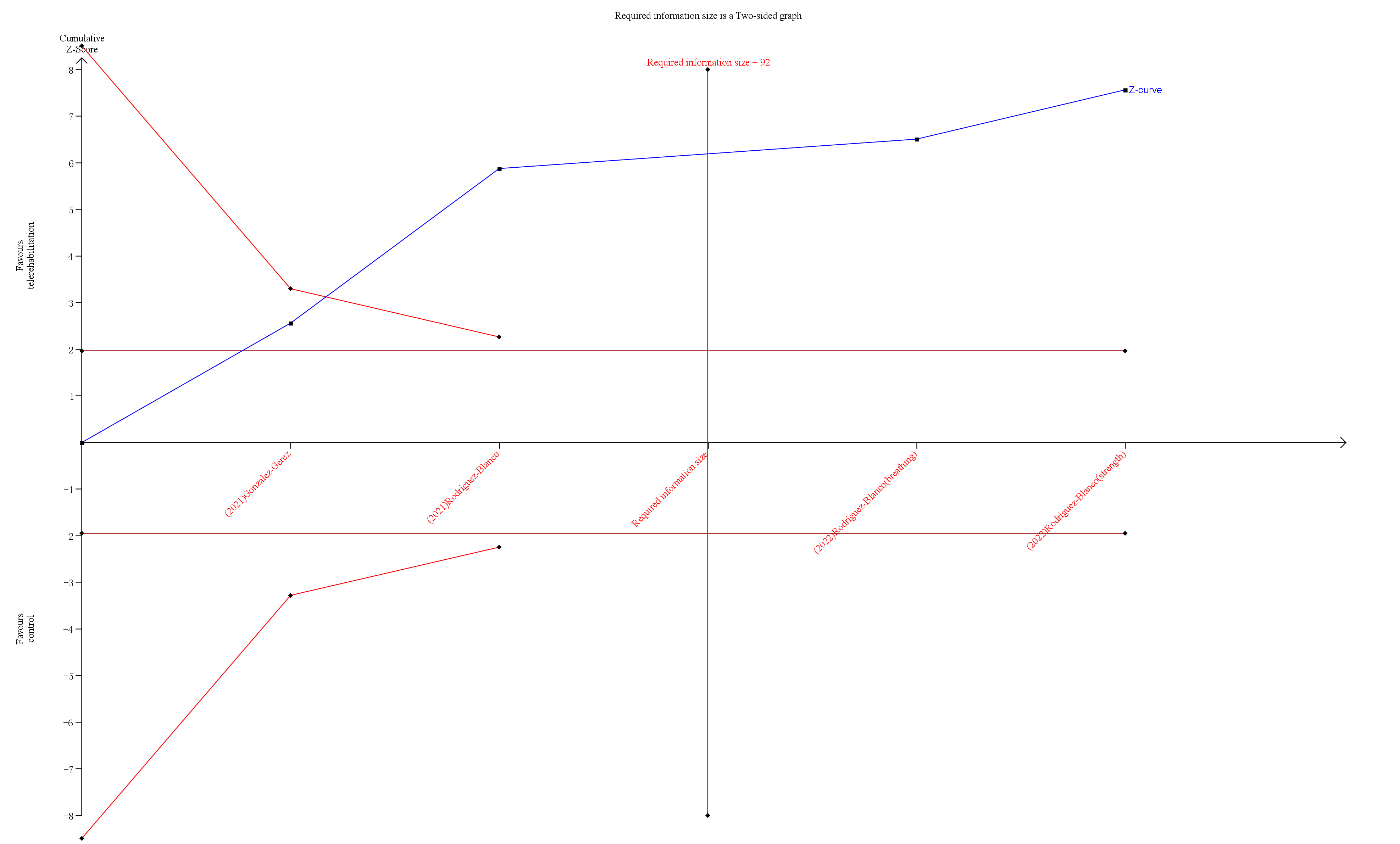


1. Supplementary Material 7B. Trial sequential analysis for Multidimensional dyspnea-12 questionnaire. Uppermost and lowermost curves represent trial sequential monitoring boundary lines (red) for benefit and harm, respectively. Horizontal lines (brown) represent the traditional boundaries for statistical significance. The cumulative Z curve (blue) crosses the monitoring boundary curve (red), indicating firm evidence that telerehabilitation is superior to no therapy or usual care.


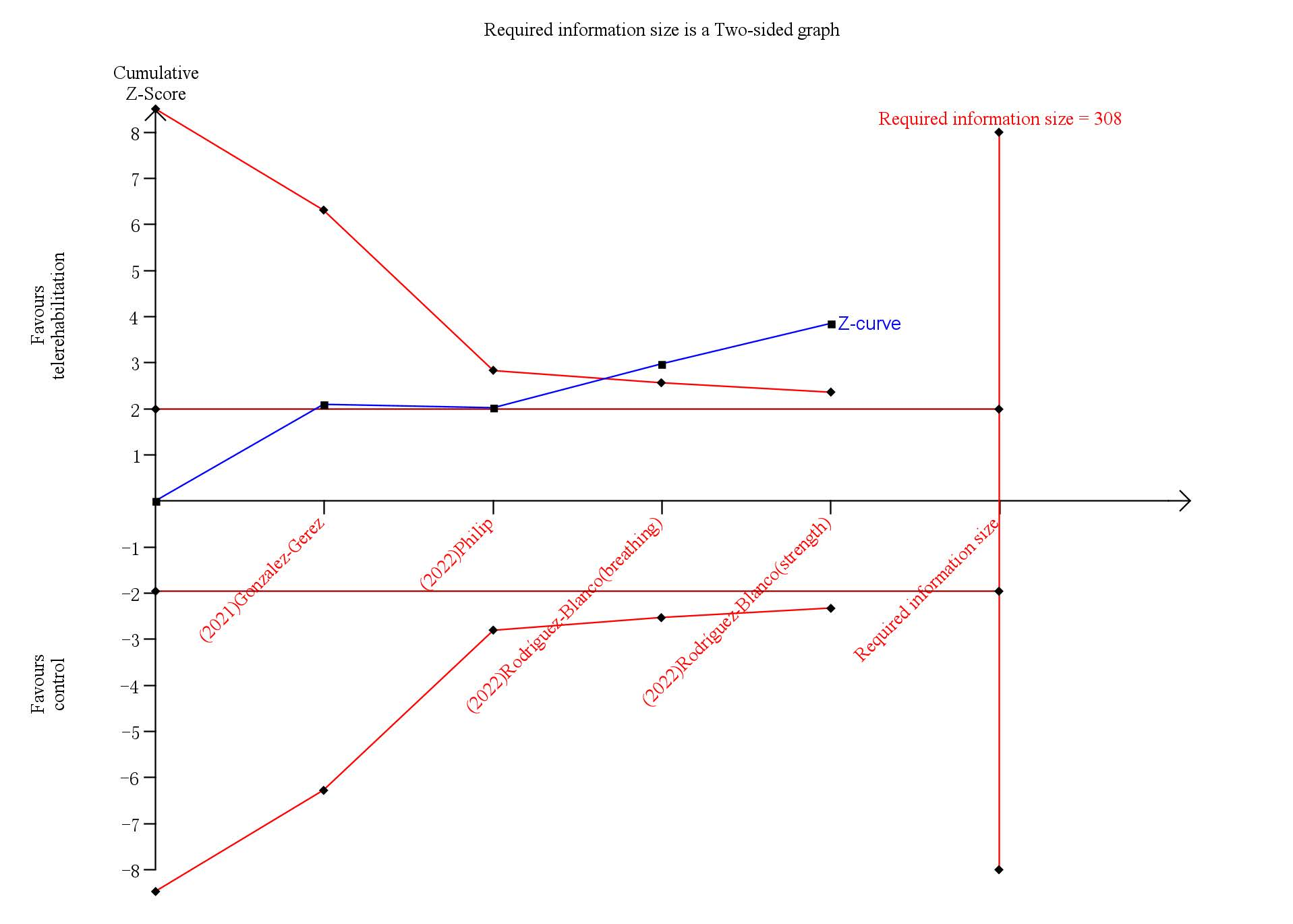


1. Supplementary Material 7C. Trial sequential analysis for 30-seconds sit-to-stand test. Uppermost and lowermost curves represent trial sequential monitoring boundary lines (red) for harm and benefit, respectively. Horizontal lines (brown) represent the traditional boundaries for statistical significance. The cumulative Z curve (blue) crosses the monitoring boundary curve (red), indicating firm evidence that telerehabilitation is superior to no therapy; meanwhile, it reached the required information size.


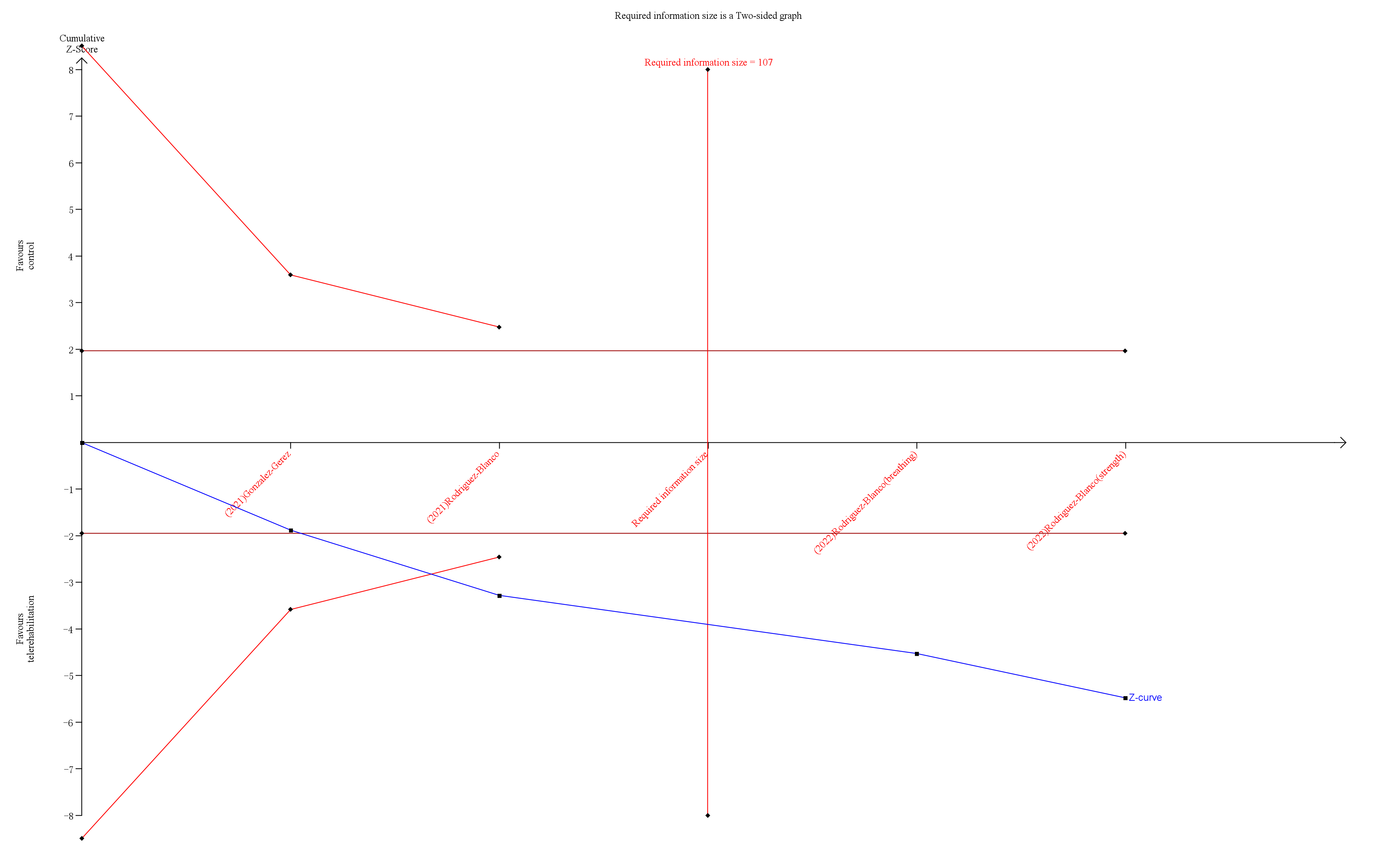


1. Supplementary Material 7D. Trial sequential analysis for Six-minute walking test. Uppermost and lowermost curves represent trial sequential monitoring boundary lines (red) for harm and benefit, respectively. Horizontal lines (brown) represent the traditional boundaries for statistical significance. The cumulative Z curve (blue) crosses the monitoring boundary curve (red), indicating firm evidence that telerehabilitation is superior to no therapy.


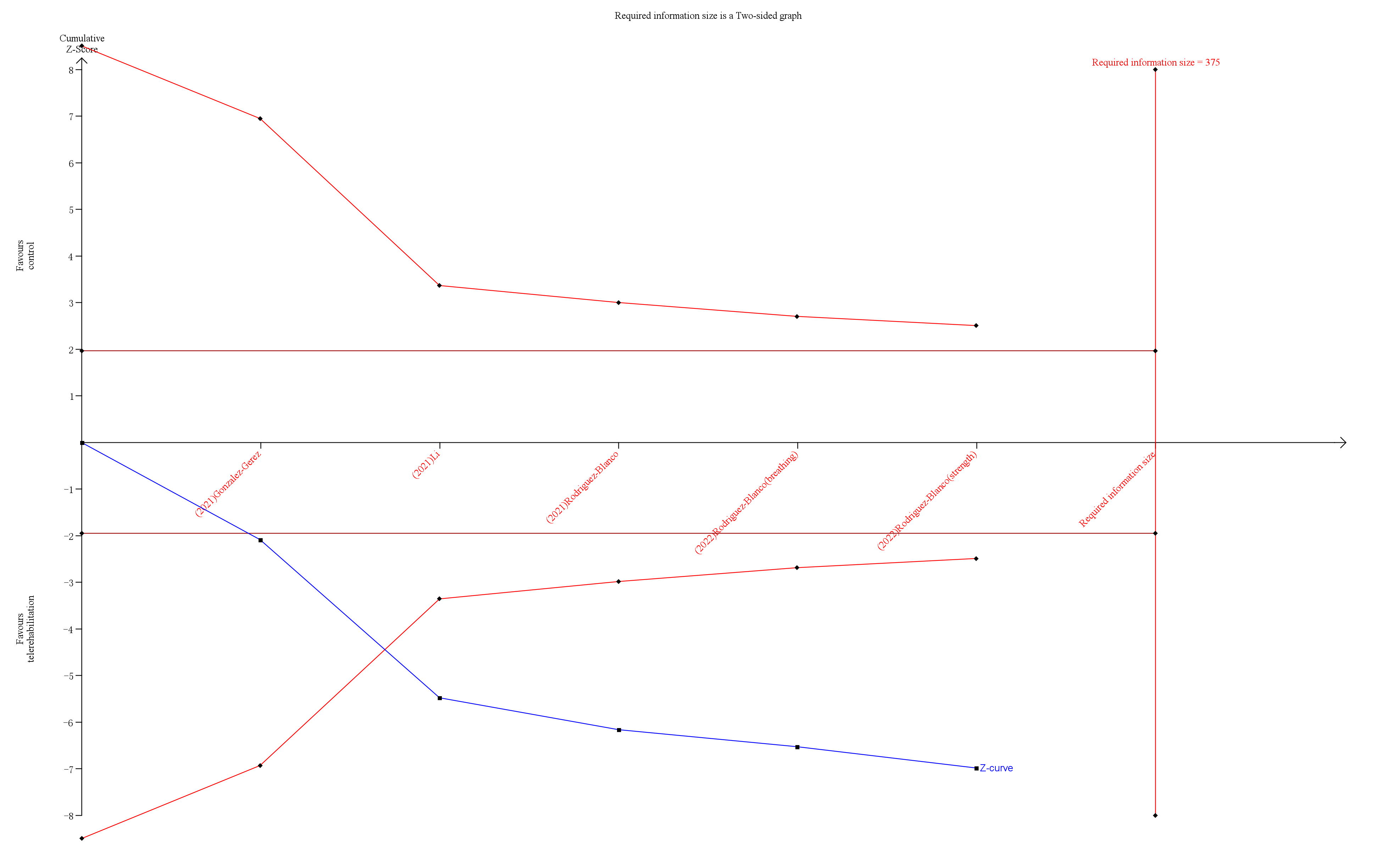


1. Multimedia Appendix 6E. Trial sequential analysis for Hamilton depression rating scale. Uppermost and lowermost curves represent trial sequential monitoring boundary lines (red) for benefit and harm, respectively. Horizontal lines (brown) represent the traditional boundaries for statistical significance. The cumulative Z curve (blue) crosses the monitoring boundary curve (red), indicating firm evidence that telerehabilitation is superior to no therapy or usual care.


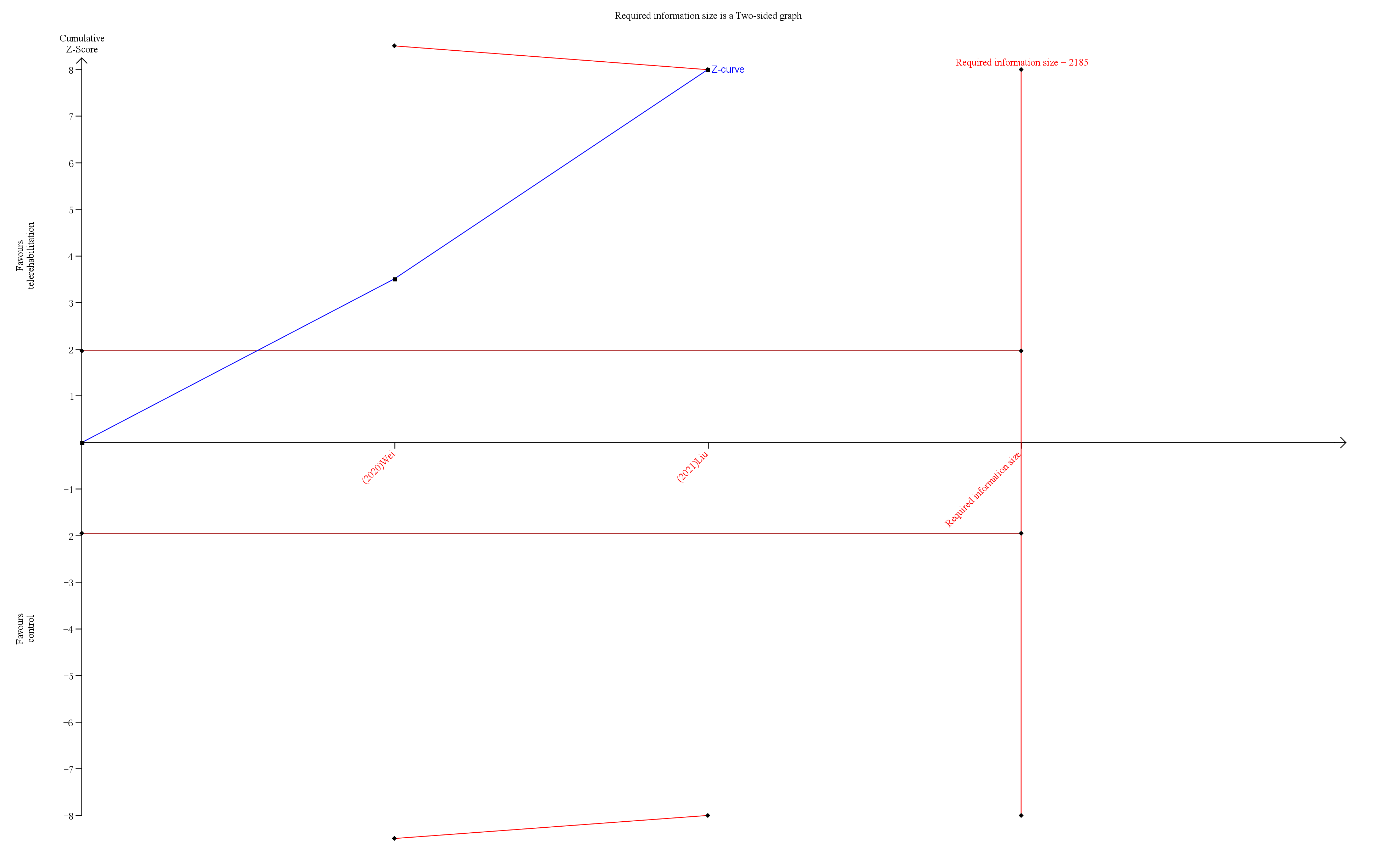

Supplement: Supplementary file 1 [file Table_1.DOCX]
